# A Meta-Analysis of Emotional Evidence for the Biophilia Hypothesis and Implications for Biophilic Design

**DOI:** 10.3389/fpsyg.2022.750245

**Published:** 2022-05-27

**Authors:** Jason S. Gaekwad, Anahita Sal Moslehian, Phillip B. Roös, Arlene Walker

**Affiliations:** ^1^Live+Smart Research Laboratory, School of Architecture and Built Environment, Faculty of Science, Engineering and Built Environment, Deakin University, Geelong, VIC, Australia; ^2^School of Architecture and Built Environment, Faculty of Science, Engineering and Built Environment, Deakin University, Geelong, VIC, Australia; ^3^School of Psychology, Faculty of Health, Deakin University, Geelong, VIC, Australia

**Keywords:** human-nature interaction, connection to nature, happiness, nature, natural environment

## Abstract

The biophilia hypothesis posits an innate biological and genetic connection between human and nature, including an emotional dimension to this connection. Biophilic design builds on this hypothesis in an attempt to design human-nature connections into the built environment. This article builds on this theoretical framework through a meta-analysis of experimental studies on the emotional impacts of human exposure to natural and urban environments. A total of 49 studies were identified, with a combined sample size of 3,201 participants. The primary findings indicated that exposure to natural environments had a medium to large effect on both increasing positive affect and decreasing negative affect. This finding supported the anticipated emotional dimension of the biophilia hypothesis and lends credibility to biophilic design theory. Evidence was revealed in support of the affective/arousal response model. Immersion in environments indicated a larger effect size than laboratory simulation of environments. Methodological recommendations for future experimental research were few, however the Positive and Negative Affect Schedule (PANAS) outcome measure was recommended as a measure of both positive and negative affect for further studies. A combination measurement of stress related outcome variables was proposed to further explore the affective/arousal response model and its potential relationship to the biophilia hypothesis. The meta-analysis provides evidence for fundamental theories regarding human-nature connection, while revealing gaps in current knowledge.

## 1. Introduction

Nature has been evidenced to provide benefits to humans who experience it (Berto, 2014; Lothian, 2017). A broad range of effects have been studied, with physiological and psychological effects the focus of the majority of research (Bowler et al., 2010). The biophilia hypothesis posits that connection with nature is beneficial to all humans, through a dominant genetic/evolutionary basis (Wilson, 1986). Furthermore, the emerging discipline of biophilic design (Kellert et al., 2007; Kellert and Calabrese, 2015) attempts to promote a human connection to anthropogenic environments through the addition of elements of nature (Kellert et al., 2007). In the same context, restorative environments design attempts to provide environments that are beneficial for human health and wellbeing, through the use of specific environmental properties as part of a design response (Kaplan, 1995; Scopelliti et al., 2019).

The present study elaborates on investigations of the relationships between humans and nature by presenting the results of a meta-analysis of experimental literature related to the relationship between exposure to natural and urban environments, and human affect. The results of this meta-analysis provide further insight into the relative benefits of nature for humans, as well as the emotional dimension of the biophilia hypothesis (Wilson, 1986), and possible directions for the future of biophilic design.

## 2. Background

### 2.1. The Biophilia Hypothesis

#### 2.1.1. Overview and Biological Basis

The term “biophilia” was first used by psychoanalyst Erich Fromm as “the passionate love of life and of all that is alive” (Fromm, 1973, p. 365). Wilson (1986) used a more specific definition, the “innate tendency to focus on life and life-like processes” (Wilson, 1986, p. 1). This focus on life is proposed to be a psychological and emotional connection that elicits complex behaviors (Kellert and Wilson, 1993).

Tidball (2012), in their review of the biophilia hypothesis (Kellert and Wilson, 1993), highlights that biophilia consists of two components. Firstly, that humans have an “affinity for other living things”, and that this affinity is “rooted in our biology” (Tidball, 2012, p. 6). The biological connection between humans and nature proposed by the biophilia hypothesis may be biologically present in human genes (Kellert and Wilson, 1993), with such a proposition developed through the idea of biocultural evolution (Lumsden and Wilson, 1985) and adaptive evolution with our ancestral environments (Tooby and Cosmides, 1990). As human evolution occurred through interaction with an environment solely comprised of the natural world, it is proposed that all humans carry a biological-based biophilic tendency (Kellert and Wilson, 1993) or have otherwise been biologically prepared to have biophilic tendencies (Seligman, 1971; Dunlap and Stephens, 2014). Such thinking includes concepts such as the savannah hypothesis (Rabinowitz and Coughlin, 1970; Orians, 1980; Balling and Falk, 1982) and prospect-refuge theory (Appleton, 1975) as well as broader concepts such as stress recovery theory (Ulrich et al., 1991), habitat theory (Appleton, 1975) and restorative environments, which substantially draws from Attention Restoration Theory (Kaplan and Kaplan, 1989).

As discussed in Han (2001) both Attention Restoration Theory (ART) and Stress Recovery Theory (SRT) are based on an evolutionary and therefore fundamentally biological framework (Kaplan and Kaplan, 1989; Ulrich et al., 1991). Therefore, not only are the two theories readily integrated by Han (2001) using a framework of “resource insufficiency”, but the integrated theory of attention and stress effects of nature on humans could be viewed as one of several “pillars” of the overarching biophilia hypothesis. This consideration may also apply to the several theories encompassing interaction between humans and nature, as previously discussed and further elaborated in the Section 2.3 of this article.

Furthermore, the biological basis of the biophilia hypothesis may be an emanation of the Biological Attraction Principle (Agnati et al., 2009). This principle, introduced in Agnati et al. (2009), suggests that there is an inherent attraction between biological systems, and “this biological attractive force is intrinsic to living organisms and manifests itself through the propensity of any living organism to act, without necessarily any direct contact, on other living organisms” (Agnati et al., 2009, p. 554).

As discussed above, the body of literature is explicit regarding the biological basis of the biophilia hypothesis. However, some effect of social/cultural mediation is acknowledged. In particular, Soule, in Kellert and Wilson (1993) acknowledges biophilia as being a complex phenomenon, which is inherent in our biology but also affected by social and cultural differences (Kellert and Wilson, 1993, p. 443). Kahn (1997) also recognizes this aspect of the biophilia hypothesis.

#### 2.1.2. Criticism and Theoretical Structure

It is worth noting that the concept of biophilia is considered to be a hypothesis. This is demonstrated by Edward Wilson when he opens the first chapter of (Kellert and Wilson, 1993), with the statement “biophilia, if it exists, and I believe it exists…” (Kellert and Wilson, 1993, p. 31). As such the hypothesis is open to challenge, evolution, and misrepresentation. There has only been sparse critical analysis of the biophilia hypothesis. A critical examination of the hypothesis was conducted by Joye and De Block (2011). Through studying the semantics of the wording of the biophilia hypothesis, Joye and De Block (2011) conclude that the definition is too broad. The outcome of the analysis results in the biophilia hypothesis being redefined by Joye and De Block (2011) as “a set of genetic predispositions of different strength, involving different sorts of affective states toward different kinds of life-like things” (Joye and De Block, 2011, p. 193). This broad general statement caters to many and conflicting interpretations, which results in the concept of biophilia being difficult to define and therefore difficult to refute.

However, as discussed above, the biophilia hypothesis itself may be necessarily broad due to the varying complexity of interactions between humans and nature. Pathways through which the biophilia hypothesis may be realized, such as an integrated SRT/ART theory (Han, 2001), may form the basis of gathering an evidence basis to support or refute the more general biophilia hypothesis.

Notwithstanding the ongoing discussion regarding the nature of biophilia, the concept forces a comparison between human-developed environments and the natural environment(s) in which humans evolved. A discrepancy is observed in the lack of ability of human-developed environments to fulfil our biological need to affiliate with life (Ministerie van Volksgezondheid, 2004; van den Berg et al., 2007). Fulfilment of this need is proposed by the biophilia hypothesis to have emotional benefits (Wilson, 1986), but as discussed above and reviewed in Bowler et al. (2010) there are a wider range of benefits to human connection with nature. The emotional effects of exposure to nature are revealed by the meta-analysis of McMahan and Estes (2015), which the present study builds on and refines concerning the biophilia hypothesis, biophilic design, and ecopsychological theory.

The scope of this paper considers an emotional relationship between humans and nature as one of the pathways through which the effects of the biophilia hypothesis are realized. The basis of this perspective is best articulated by Wilson: “Biophilia…is the innately emotional affiliation of human beings to other living organisms” (Kellert and Wilson, 1993, p. 31). It is acknowledged that there are other avenues through which the effects of the human nature interaction may be realized, which may include attention and stress as previously discussed.

### 2.2. Biophilic Design

Biophilic design is “the deliberate attempt to translate an understanding of the inherent human affinity with natural systems and processes—known as biophilia—into the design of the built environment” (Kellert et al., 2007, p. 3). Biophilic design intends to reconcile the occupants of the built environment with the life and life-like processes that are present in natural environments. This is in contrast with the development of the contemporary built environment, which degrades natural systems and isolates its occupants from the natural environment (Grahn and Stigsdotter, 2010).

Biophilic design theory was proposed in its current form by Kellert et al. (2007) and further developed by many, including Browning et al. (2014); Kellert and Calabrese (2015); Salingaros (2015); Downton et al. (2017). It is recognized that many of the ideas present in biophilic design theory were pre-existing, including psychological concepts such as restorative environments (Ulrich, 1981; Kaplan and Kaplan, 1989), prospect/refuge theory (Appleton, 1975), and concepts from landscape architecture such as the “six sublimities” of traditional East Asian Garden design (Suthasupa, 2012).

### 2.3. Theoretical Landscape

Several theories consider the connection between humans and nature, either exclusively or as part of a broader hypothesis. These theories have generally been developed through the disciplines of ecopsychology (Hasbach and Kahn, 2012) and environmental psychology (Steg and de Groot, 2019). Such theories include those conveniently grouped by Albrecht (2019) under the umbrella term “psychoterratic typologies”, a term encompassing the relationship between the human psyche and the earth. There are a number of both positive and negative human-earth affiliations considered under this term. Notable affiliations include topophilia (connection to place) (Tuan, 1990), ecophilia (connection to ecosystem) (Sobel, 1996), and biophilia (connection to nature) (Wilson, 1986). The concept of topophilia was explored by Tang et al. (2015) and Berto et al. (2018) as part of research in biophilia through the concept and effects of familiarity with place.

The relationship between the biophilia hypothesis and ecopsychology is made apparent by Roszak et al. (1995):

“in a sense, ecopsychology might be seen as a commitment by psychologists and therapists to the hope that the biophilia hypothesis will prove true and so become an integral part of what we take mental health to be” (Roszak et al., 1995, p. 4)

From an ecopsychological perspective, the connection between humans and nature goes beyond the mere visual or aesthetic dimensions. Yet, perhaps from the domination of the visual sense, most literature focus on the visual aspects of natural environments and its affects. Humans are multisensory beings, thus the benefits from sensory experiences in health and wellbeing are considered at a multisensory scale and dimension (Franco et al., 2017).

In summary, the biophilia hypothesis forms part of a body of theories that address the relationship between humans and nature. The biophilia hypothesis is considered to have a biological basis. However, it is also argued that behaviors of positive human-nature interactions are partially inherited by natural selection, and partially by learned through cultural evolution (Sideris, 2003; Tidball, 2012).

The meta-analysis presented herein studies the emotional dimension of the biophilia hypothesis only, but acknowledges that there are other dimensions through which biophilia may be realized. The present work provides insight not only into the validity of the biophilia hypothesis having emotional benefits to humans, but the properties of exposure to nature which may elicit stronger positive reactions, such as level of sensory connection. Further, as noted previously, the experiences of nature at a psychological and physical level are at a multisensory dimension that includes aspects beyond the visual experience. These insights also have implications for biophilic design practice.

## 3. Objectives

The primary objective of this meta-analysis was developed using a population, exposure, comparison, outcome (PECO) (Morgan et al., 2018) format. The research question was, “In adults, is immersive or laboratory exposure to natural environments effective, compared to equivalent exposure to urban environments, in increasing positive emotional state and decreasing negative emotional state?”. This question was used to formulate the inclusion criteria of the literature search. It was hypothesized that exposure to natural environments would benefit the affective state of participants. This hypothesis aligned with the biophilia hypothesis.

The two further objectives of the study investigated the causes of potential effects. The first further objective was to determine the relative effects of immersion in environments as compared to laboratory simulation of environments. This objective provides insight into whether an increase in sensory connection to an environment results in increased benefits from experiencing that environment. This information may be used to design more effective experimental procedures. It was hypothesized that an increasing level of sensory connection to the environment would increase the emotional effect of that environment.

The second further objective concerned an investigation of the relative effect of a range of experimental methods in testing the effects of test environment on affect. This objective is similarly concerned with advising on specific experimental methods for future testing of the biophilia hypothesis. This objective was exploratory and as such, an explicit hypothesis was not considered.

The significance of the meta-analysis of positive and negative affect aligns with an exploration of the biophilia hypothesis. That is, evidence to support the hypothesis of this study serves to support the biophilia hypothesis. Furthermore, quantitative validation of the effects of nature and exploration of the methods used to determine these effects may be used by researchers testing the effectiveness of nature exposure on human emotions.

## 4. Methods

### 4.1. Inclusion Criteria

The inclusion criteria directly addressed the primary question of the review discussed in the Objectives section. The criteria were initially determined as part of a PECO (Morgan et al., 2018) framework, with further criteria added. Detailed article content inclusion criteria for this review were as follows:

- Study subjects/population: Adults (over the age of 18). Participants without specific mental or physical health conditions. Studies on specific populations (e.g., patients in hospitals, children in schools) were excluded in order to preserve a degree of external validity to the results of the meta-analysis. Similarly, stimuli that are specific to a population group (view from the classroom, view from the nurses' station, view from inpatient ward windows, etc.) were excluded.
- Type of exposure: Immersive or laboratory simulation of environments. Laboratory simulation must have included visual simulation as a minimum (photograph slides, video) but may have included other sensory simulation also (e.g., audio simulation). Studies that used immersive virtual reality environments or non-visual senses in isolation (e.g., audio only) were excluded. Immersion was defined as viewing an environment from within that environment (e.g., sitting on a chair within a city park). If exercise (such as a walk through a park) formed part of the exposure, it must have been applied to both the nature intervention and urban comparator.
- Type of comparator: Studies must have featured equivalent exposure to both natural and urban environments. The classification of these two environments must have been discussed in-text. Studies which inferred these environments from other variables were excluded (e.g., level of “nature” cannot have been inferred from leaf area index of a suburban area).
- Type of outcome: Emotional or affective state must have been reported as an outcome measure.
- Type of article: Published, peer reviewed experimental studies in English were included. Reviews, book chapters, conference papers, non-English records, and duplicate entries were excluded. No date criteria were applied.
- Type of study: Between-subject or within-subjects randomized experimental trials were included. The statistical analysis and presentation of results in the articles must have had controlled for environment as an independent variable.

### 4.2. Literature Search

Two authors conducted a systematic review of the literature to identify relevant studies which experimentally investigated the relationship between exposure to natural and urban environments, and both positive and negative affect.

The literature search was conducted in November 2021. Six databases were searched, encompassing general, psychological, and health related fields. The databases searched were Scopus, Web of Science, PsycINFO, Global Health, Medline, and CINAHL Complete. The latter three databases were searched through the EBSCOhost platform. This search strategy was used to cover breadth of publications in a number of fields, as well as targeting the psychological and health disciplines where the majority of publications were expected to be found. No date filter was applied. The search strings used are presented in Supplementary Material.

Articles were also identified through a reference list review and as part of a narrative literature review. As part of the literature search, several literature review and meta-analysis articles were identified. Although these articles did not meet the inclusion criteria for the search, their reference lists were reviewed for relevant articles. Furthermore, during the full-text review of articles, in-text references relevant to the objective of the meta-analysis were also included. This second method is considered an extension of a typical narrative literature review. A total of 70 articles were added to the database search result pool of 1947 articles, for a total number of 2,017 articles identified.

**Figure 1:**
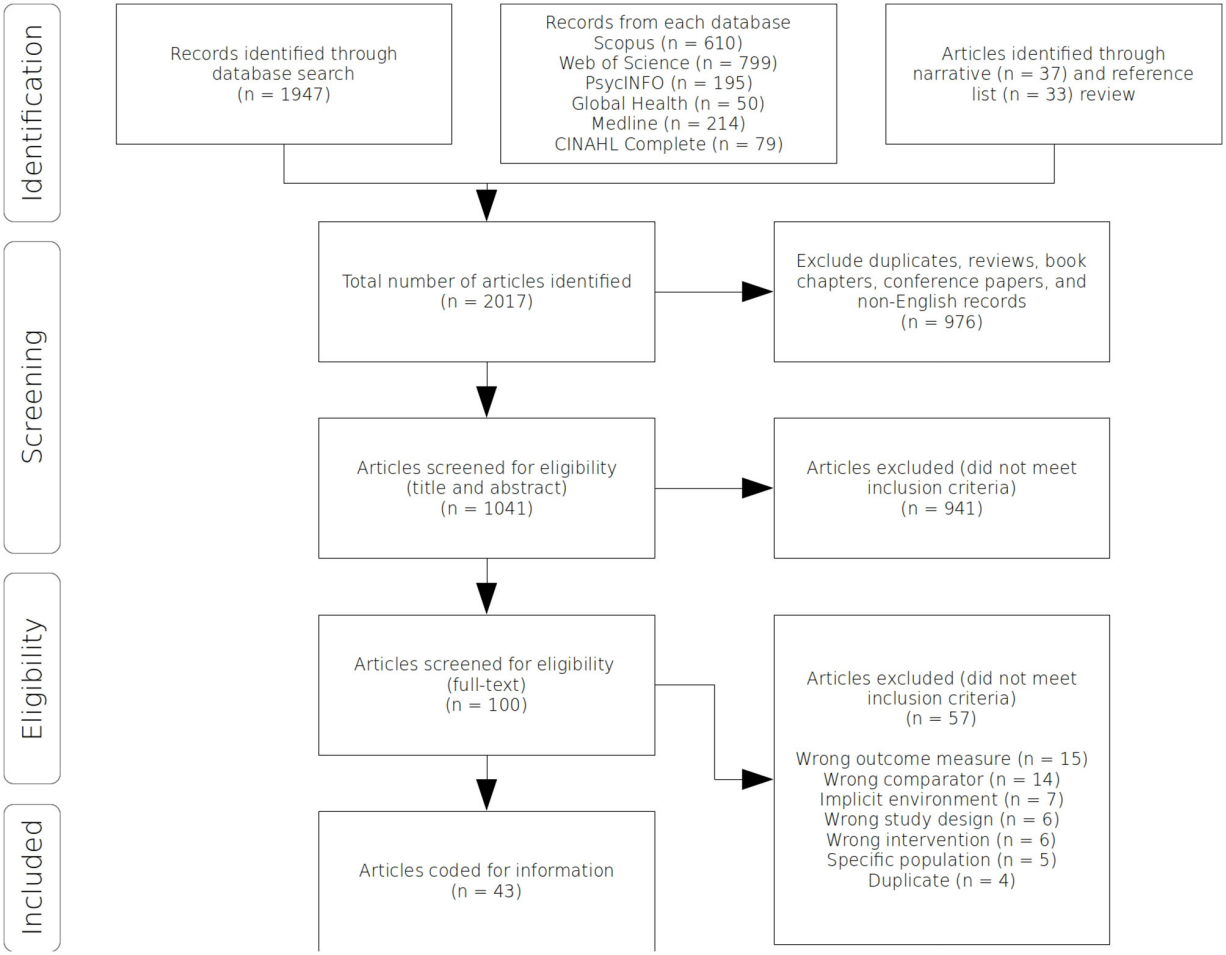
Flow diagram of the systematic review process.

The extracted article data were screened to identify relevant articles. Article metadata (citations, title, abstract) generated from each database search were imported into the proprietary “Covidence” software environment, in which the title and abstract review took place. To address potential bias in the application of inclusion criteria, two reviewers conducted the review at title and abstract stage. A Cohen's kappa statistic (McHugh, 2012) of 0.55 was calculated, with a proportionate agreement of 0.9. This value of Cohen's kappa represents the higher end of “moderate agreement” (McHugh, 2012) and is considered acceptable given the high level of heterogeneity expected between articles in the review. Disagreements between the two reviewers were discussed through notes in the Covidence software environment, with satisfactory conclusions reached for all disagreements. Following the title and abstract screening process, the full-text review was conducted by the corresponding author. Included articles were coded for metadata and experimental data. A PRISMA flow diagram of the systematic review process is presented in Figure 1.

#### 4.2.1. Article Quality

Articles were rated for quality by a single author. The work of Thomas et al. (2004) and the application of this work by Bowler et al. (2010) were used as a basis for the development of the quality criteria used for this study. Significant overlap between the recommendations of these works and the inclusion criteria of the literature search was identified. Hence, in addition to the strict inclusion criteria, a set of three quality criteria were considered sufficient to address the objectives of the meta-analysis:

- Recruitment of participants: Random recruitment was considered to be high quality. Self-selection of participants was considered to be moderate quality.
- Quality of data presented: Pre- and post- treatment mean and standard deviation data were considered the highest quality. Reported difference data were also considered to be of high quality. Post-treatment data only were considered to be of moderate quality.
- Discussion and control of potential confounding variables: Inclusion of such a discussion in a study was considered to be of high quality. No discussion was considered to be of low quality.

Studies were classified as being of low, moderate, or high quality. Only moderate and high quality studies were included in the review. Due to the strict inclusion criteria of the review, it was anticipated that few low quality articles were to be identified as part of the literature search.

#### 4.2.2. Heterogeneity

Significant heterogeneity between studies was expected, within the scope of the inclusion criteria. The potential causes for heterogeneity were as follows:

- Studies utilizing different measurement methods for emotional state
- A wide definition of what constitutes “natural” and “urban” environments, leading to varying effects of these environments on participants
- Varying methods within the definition of “immersion” and “laboratory” exposures
- Geographic or individual factors.

As discussed in the Section 3, this meta-analysis sought to explore the reasons for this heterogeneity and provide advice for future studies.

### 4.3. Data Synthesis

The data synthesis consisted of both a review of article metadata and quantitative analysis of the results reported. Article metadata were extracted for each full-text article included in the study. This metadata included study location (country), experimental procedure (between-subjects or within-subjects), participant types and recruitment method, mean age, environment exposure type (immersion or laboratory), exposure duration, use of a stressor, type of natural environment (wild or human-managed), and method used to measure emotional state. Quantitative result data was extracted from each study.

For pre/post data, the difference between the means and standard deviations was calculated to provide a measure of the difference in outcome variable that the intervention resulted in. For between-subjects designs a pooled standard deviation was used. For within-subjects designs the method to calculate standard deviations from difference scores presented in Lakens (2013) was used. This method required an assumption to be made regarding the correlation between measures. The assumption used for the data synthesis was *r* = 0.5, according to Fu et al. (2008).

Effect sizes and confidence intervals were calculated as follows. Cohen's d was used as the reported effect size (Cohen, 1988). For between-subjects designs, a pooled standard deviation was used as the denominator. For within-subjects designs, an arithmetic average of standard deviation was used as the denominator. Confidence intervals were calculated by first determining the variance of the effect size using the method of Cooper (2010) then applying this variance to determine the two-tailed 95% confidence interval. For studies that only reported a test statistic, the effect size was calculated using the methods and calculator of Lakens (2013).

Calculation of a weighted average effect size was conducted using the random-effects method presented in Borenstein (2009). This method used the method of moments to determine (T^2^). A random-effects method was considered appropriate given the variability in study methods.

Several studies reported on subscales but did not provide scale totals, for example Shin et al. (2011) reported on subscales of the Profile of Mood States (McNair et al., 1971) but did not report a total. For the purposes of the meta-analysis, each reported subscale was treated as its own study, with the calculated effect size assigned to either positive affect or negative affect depending on the function of the subscale.

## 5. Results

### 5.1. The Body of Literature

A total of 49 studies across 43 articles were identified. If an article contained reporting on more than one study which met the inclusion criteria, each included study was considered individually. Positive affect data were reported by 46 studies and 30 studies reported data on negative affect. Pre-test and post-test mean and standard deviation data were reported by 17 studies and 3 studies reported pre/post difference data. Post- data only were reported by 29 studies, either through presenting mean and standard deviation data or test statistics.

Quantitative analysis of effect sizes was conducted on two resulting data sets, a post-test cohort (29 studies) and a pre/post cohort. Post-test data from the pre/post cohort (20 studies) was combined with the post-test only cohort (20 studies) to build the combined post-test dataset of 49 studies.

### 5.2. Study Quality

No high or low quality studies were identified during the study quality assessment process. All studies were classified as being of moderate quality.

Classification of study quality was largely driven by the infrequent discussion of potential confounding effects in the reviewed articles. 10 studies discussed confounding effects, including season and environment (Brooks et al., 2017; Bielinis et al., 2018; Lopes et al., 2020; Liu et al., 2021) and background stress (Johansson et al., 2011). Bratman et al. (2015) and Golding et al. (2018) conducted comprehensive investigation into the potential confounding effects of demographics and pre-exposure outcome measures. The results of these two studies indicated that there were no confounding effects of demographic or pre-exposure outcome measures. This provides some measure of reliability to studies that did not investigate potential confounding effects.

Participant recruitment information was not satisfactorily reported for the majority of studies. University students were overwhelmingly used as participants (42 studies). The recruitment method for these students included self selection (through advertising on social media and flyers) and random recruitment from a university participant pool (considered to be random recruitment for the purposes of quality assessment). Other participant types were all self-selected, and included the general population (McAllister et al., 2017; Lopes et al., 2020) and professionals (Tyrväinen et al., 2014; Reeves et al., 2019).

Significant variation was observed regarding the quality of output results reported. Pre/post group means and standard deviations were reported by 17 studies. A further 3 studies reported on the pre/post difference. These studies allowed for the calculation of the full pre/post effect of environmental exposure to be established. Post-treatment means and standard deviations were reported by 18 studies. Without controlling for pre-treatment state, these datasets lacked enough information to determine the true effect of the exposure to environments. However, due to the prevalence of post-treatment data being reported, and the acceptable reporting quality of such data, these studies were included in the analysis. The remaining 11 studies reported statistical test figures. These were either *F*-test (ANOVA) or *t*-test results. While the *t*-test results simply compared means, a range of ANOVA dimensions were reported including between multiple environments (Ulrich et al., 1991) and time × environment interactions (Bratman et al., 2015).

### 5.3. Description of Studies

#### 5.3.1. Study Locations

The body of research indicated a strong participation from the northern hemisphere, with 45 of the 49 studies conducted in the northern hemisphere. The majority (18 studies) were conducted in the US, with the second most prevalent geography being Canada (6 studies). Other countries included the UK, China, Japan, South Korea, the Netherlands, Poland, Finland, Iceland, and Denmark.

#### 5.3.2. Experimental Method

All included studies were either of a between-subjects or within-subjects design. Considerable variation in sample sizes was identified. 29 studies testing participants by immersion in environments, and 20 studies utilized laboratory simulation of environments. The majority of studies (30 studies) utilized a between-subjects design, with 19 studies utilizing a within-subjects design.

Total sample sizes for all studies ranged from 17 (Jiang et al., 2021) to 306 (Pasca et al., 2021), generally evenly split between nature and urban groups. The total number of participants across all studies was 3201. The median total sample size was 54 participants.

#### 5.3.3. Environments

Natural study environments were classified as either being created by humans (“human-managed”) or not being created by humans (“wild”). Wild environments were typically national parks and state forests, whereas managed environments were typically urban parks. It is recognized that the wild environments discussed herein are maintained due to considerable management from humans. However, the terminology is used as a convenient distinction between types of natural environments. This broad definition of what may constitute “nature” is consistent with the proposition of Bratman et al. (2012), that nature is “areas containing elements of living systems that include plants and non-human animals across a range of scales and degrees of human management, from a small urban park to a relatively pristine wilderness” (Bratman et al., 2012, p. 120). Urban environments were not classified as part of the analysis.

A total of 22 studies exposed participants to wild environments only and 25 studies exposed participants to human-managed environments only. From the information available on each study (sample images of test environments) the spatial extent of the managed environments appears to have allowed immersion without distraction by other environments. In general, limited information about environments was provided beyond environment location and one or two images per study. Hence, the potential for environmental interference (e.g., traffic noise, visibility of tall buildings) was unknown for all studies. Participants were exposed to both wild and human-managed environments in two studies.

A summary of studies and associated metadata is provided in Table 1.

**Table 1:** Summary of metadata from experimental studies reporting on the relative effect of exposure to natural and urban environments on positive and negative affect.

**Study**	**Country**	**Study design**	**Exposure duration**	**Mean age**	**Exp. type**	**Env. type**	**Activity**
Berman et al. (2008) Study 1	US	Within	50 to 55 min	22.62	I	M	W
Berman et al. (2008) Study 2	US	Within	10 min	24.25	L	W	S
Hartig et al. (1991) Study 2	US	Between	40 min	20	I	M	W
Hartig et al. (1991) Study 2	US	Between	40 min	20	I	M	W
Hartig et al. (1999) Study 1	US	Between	8 min	20.1	L	W	S
Hartig et al. (2003)	US	Between	50 min	20.8	I	W	W
Ryan et al. (2010) Study 2	US	Between	15 min	20	I	M	W
Ryan et al. (2010) Study 3	US	Between	8 min	20	L	W	S
Sheets and Manzer (1991) Study 1	US	Between	-	-	L	M	S
Sheets and Manzer (1991) Study 2	US	Between	-	-	L	M	S
Ulrich et al. (1991)	US	Between	10 min	-	L	W	S
Ulrich et al. (1991)	US	Between	10 min	-	L	W	S
Ulrich et al. (1991)	US	Between	10 min	-	L	W	S
Ulrich et al. (1991)	US	Between	10 min	-	L	W	S
Ulrich et al. (1991)	US	Between	10 min	-	L	W	S
Mayer et al. (2009) Study 1	US	Between	10 min walk, 5 min sit	-	I	W	W, S
Mayer et al. (2009) Study 2	US	Between	10 min walk, 5 min sit	-	L	M	S
Nisbet and Zelenski (2011) Study 1	Canada	Between	17 min	20.8	I	M	W
Nisbet and Zelenski (2011) Study 2	Canada	Between	17 min	-	I	M	W
Tennessen and Cimprich (1995)	US	Between	-	20	L	M	S
Tennessen and Cimprich (1995)	US	Between	-	20	L	M	S
White et al. (2010) Study 1	England	Within	-	28.5	L	W	S
Reeves et al. (2019)	UK	Within	10 min	41	I	M	S
Stewart and Haaga (2018)	International	Between	15 min	-	L	W	S
Golding et al. (2018)	UK	Between	5 min	27	L	W	S
McAllister et al. (2017)	Australia	Between	2 min 30 s	49.07	L	W	S
McAllister et al. (2017)	Australia	Between	2 min 30 s	49.07	L	W	S
Lee et al. (2009)	Japan	Within	15 min, 3 sessions	21.3	I	W	S
Park et al. (2007)	Japan	Within	20 min walk, 20 min sit	22.8	I	W	W, S
Park et al. (2007)	Japan	Within	20 min walk, 20 min sit	22.8	I	W	W, S
van den Berg et al. (2003)	Netherlands	Between	7 min	21.9	L	W	S
van den Berg et al. (2003)	Netherlands	Between	7 min	21.9	L	W	S
van den Berg et al. (2003)	Netherlands	Between	7 min	21.9	L	W	S
van den Berg et al. (2003)	Netherlands	Between	7 min	21.9	L	W	S
Tyrväinen et al. (2014)	Finland	Within	30 min	47.64	I	Both	W
Tyrväinen et al. (2014)	Finland	Within	30 min	47.64	I	Both	W
Olafsdottir et al. (2018)	Iceland	Between	40 min	24.39	I	W	W
Olafsdottir et al. (2018)	Iceland	Between	40 min	24.39	I	W	W
Stigsdotter et al. (2017)	Denmark	Within	15 min	-	I	W	W
Stigsdotter et al. (2017)	Denmark	Within	15 min	-	I	W	W
Stigsdotter et al. (2017)	Denmark	Within	15 min	-	I	W	W
Stigsdotter et al. (2017)	Denmark	Within	15 min	-	I	W	W
Stigsdotter et al. (2017)	Denmark	Within	15 min	-	I	W	W
Stigsdotter et al. (2017)	Denmark	Within	15 min	-	I	W	W
Stigsdotter et al. (2017)	Denmark	Within	15 min	-	I	W	W
Brown et al. (2013)	UK	Within	10 min, 2 sessions	36.91	L	W	S
Shin et al. (2011)	South Korea	Within	50 to 55 min	23.27	I	M	W
Shin et al. (2011)	South Korea	Within	50 to 55 min	23.27	I	M	W
Shin et al. (2011)	South Korea	Within	50 to 55 min	23.27	I	M	W
Shin et al. (2011)	South Korea	Within	50 to 55 min	23.27	I	M	W
Shin et al. (2011)	South Korea	Within	50 to 55 min	23.27	I	M	W
Shin et al. (2011)	South Korea	Within	50 to 55 min	23.27	I	M	W
Beute and de Kort (2014) Study 2	Netherlands	Between	45 min	21.1	L	W	S
Bielinis et al. (2018)	Poland	Between	15 min, 2 sessions	21.4	I	M	S
Bielinis et al. (2018)	Poland	Between	15 min, 2 sessions	21.4	I	M	S
Bielinis et al. (2018)	Poland	Between	15 min, 2 sessions	21.4	I	M	S
Bielinis et al. (2018)	Poland	Between	15 min, 2 sessions	21.4	I	M	S
Bielinis et al. (2018)	Poland	Between	15 min, 2 sessions	21.4	I	M	S
Bielinis et al. (2018)	Poland	Between	15 min, 2 sessions	21.4	I	M	S
Bielinis et al. (2018)	Poland	Between	15 min, 2 sessions	21.4	I	M	S
Geniole et al. (2016)	Canada	Within	15 min, 2 sessions	24.6	I	M	W
Janeczko et al. (2020)	Poland	Between	30 min	-	I	W	W
Johansson et al. (2011)	Sweden	Within	40 min, 4 sessions	23.3	I	M	W
Johansson et al. (2011)	Sweden	Within	40 min, 4 sessions	23.3	I	M	W
Liu et al. (2021)	China	Within	30 min	-	I	W	S
Liu et al. (2021)	China	Within	30 min	-	I	W	S
Liu et al. (2021)	China	Within	30 min	-	I	W	S
Liu et al. (2021)	China	Within	30 min	-	I	W	S
Liu et al. (2021)	China	Within	30 min	-	I	W	S
Liu et al. (2021)	China	Within	30 min	-	I	W	S
Lopes et al. (2020)	Portugal	Between	30 min	25	I	M	W
Neale et al. (2021) Study 1	US	Within	400 s	20	L	W	S
Takayama et al. (2014)	Japan	Within	15 min	20	I	M	W
Takayama et al. (2014)	Japan	Within	15 min	20	I	M	W
Takayama et al. (2014)	Japan	Within	15 min	20	I	M	W
Takayama et al. (2014)	Japan	Within	15 min	20	I	M	W
Takayama et al. (2014)	Japan	Within	15 min	20	I	M	W
Takayama et al. (2014)	Japan	Within	15 min	20	I	M	W
Takayama et al. (2014)	Japan	Within	15 min	20	I	M	W
Brooks et al. (2017) Study 1	Canada	Between	10 min, 2 sessions	21.5	I	M	W
Brooks et al. (2017) Study 2	Canada	Between	15s per slide	21.3	L	M	S
Jiang et al. (2021)	International	Between	Less than 20 min	-	L	M	S
Lee et al. (2015a)	Japan	Within	15 min, 2 sessions	22.3	I	M	S
Lee et al. (2015a)	Japan	Within	15 min, 2 sessions	22.3	I	M	S
Lee et al. (2015a)	Japan	Within	15 min, 2 sessions	22.3	I	M	S
Lee et al. (2015a)	Japan	Within	15 min, 2 sessions	22.3	I	M	S
Lee et al. (2015a)	Japan	Within	15 min, 2 sessions	22.3	I	M	S
Lee et al. (2015a)	Japan	Within	15 min, 2 sessions	22.3	I	M	S
Navalta et al. (2021)	US	Within	30 min	29.2	I	M	S
Navalta et al. (2021)	US	Within	30 min	29.2	I	M	S
Nisbet et al. (2019)	Canada	Between	30 min	19.58	I	M	W
Pasca et al. (2021)	International	Between	2 min 30 s	20.48	L	W	S
Song et al. (2014)	Japan	Within	15 min	21.2	I	M	W
Song et al. (2014)	Japan	Within	15 min	21.2	I	M	W
Song et al. (2014)	Japan	Within	15 min	21.2	I	M	W
Song et al. (2014)	Japan	Within	15 min	21.2	I	M	W
Song et al. (2014)	Japan	Within	15 min	21.2	I	M	W
Song et al. (2014)	Japan	Within	15 min	21.2	I	M	W
Song et al. (2014)	Japan	Within	15 min	22.5	I	M	W
Ballew and Omoto (2018)	US	Between	15 min	19.3	I	Both	S
Bratman et al. (2015)	US	Between	50 min	22.9	I	M	W
Pretty et al. (2005)	UK	Between	20 min	24.6	L	W	W
Pretty et al. (2005)	UK	Between	20 min	24.6	L	W	W
Pretty et al. (2005)	UK	Between	20 min	24.6	L	W	W
Pretty et al. (2005)	UK	Between	20 min	24.6	L	W	W
Pretty et al. (2005)	UK	Between	20 min	24.6	L	W	W
Pretty et al. (2005)	UK	Between	20 min	24.6	L	W	W
Koselka et al. (2019)	US	Within	50 min, 4 sesssions	22.62	I	M	W

*Dash(–) indicates information not reported. Exp. Type, Exposure Type. For Exposure Type: I, Immersion; L, Laboratory Simulation. Env. Type, Environment Type. For Environment Type: M, Managed; W, Wild. For Activity: W, Walking, S, Sitting*.

#### 5.3.4. Outcome Measures

Studies were classified based on the use of outcome measures. Both evidenced tools and custom, often single item scales were used to measure outcomes. The majority of studies (23 studies) used the Positive and Negative Affect Schedule (PANAS) (Watson et al., 1988) to measure both positive and negative affect. However, 3 studies omitted PANAS results for negative affect. The Zuckermann Inventory of Personal Reactions (ZIPERS) (Zuckerman, 1977) was used by 4 studies. All 4 of these studies were conducted by the same two lead authors. Ulrich et al. (1991) provided data on each of the ZIPERS factors whereas only the total ZIPERS results were provided for the other 3 studies (Hartig et al., 1991, 1999, 2003). All studies which used the ZIPERS measures reported test statistics only in their results, and did not provide pre/post mean and standard deviation data. Ten studies used the Profile of Mood States (POMS) (McNair et al., 1971) to measure affective disturbance. POMS scores were provided as totals by Tennessen and Cimprich (1995) and Stigsdotter et al. (2017). The article b van den Berg et al. (2003) provided results for 3 of the 6 factors only. All other studies which utilized the POMS scale provided data on the 6 factors, but not total figures. The Subjective Vitality Scale (SVS) was used by 3 studies.

Other studies used a range of measures, generally single-item metrics to measure factors such as happiness (Hartig et al., 1991; van den Berg et al., 2003; White et al., 2010), subjective comfort (Park et al., 2007; Lee et al., 2009), subjective calm (Park et al., 2007), affective pleasure (Sheets and Manzer, 1991), and positive emotions (Ballew and Omoto, 2018).

### 5.4. Data Synthesis

#### 5.4.1. Consideration of Publication Bias

The possibility of publication bias was assessed using three methods. The first was through examining the symmetry of funnel plots (Borenstein, 2009), displayed as effect size plotted against Precision (1 / Standard Error) (Egger et al., 1997). Funnel plots for positive and negative affect displayed generally symmetrical behavior, indicating a low likelihood of publication bias. The regression test of Egger et al. (1997) was used to quantify potential asymmetry in the funnel plots. This test indicated potential asymmetry in both the positive and negative affect datasets. A trim and fill analysis (Borenstein, 2009) was then undertaken to determine an adjusted effect size, accounting for publication bias. Both estimators *L*_0_ and *R*_0_ indicated that the article dataset did not need to be trimmed. This result concurred with the graphical interpretation of the funnel plots but disagreed with the results of Egger's test (Egger et al., 1997). The large effect size of Pasca et al. (2017) may have skewed the regression fit of Egger's test for both positive and negative affect results. However, upon further inspection of Pasca et al. (2017) no valid reason for excluding the data points from the meta-analysis work could be determined. Hence, the outlying data points of Pasca et al. (2017) were retained as part of the dataset, and it was concluded that publication bias did not have a significant effect on the meta-analysis dataset. No further corrections to the dataset were undertaken.

#### 5.4.2. Post-treatment Dataset

Effect sizes were calculated for all studies with the variance of effect size used to calculate the 95% confidence interval of effect size. These results are presented in Table 2. As previously discussed in the Section 4, a random-effects meta-analysis method was used to combined study effect size data into a single weighted average figure. Combined effect sizes and 95% confidence interval of effect size for various data subsets are presented in Table 3.

**Table 2:** Summary of effect size data from experimental studies reporting on the relative effect of exposure to natural and urban environments on positive and negative affect.

**Study**	***N***	**Outcome measure**	**Pos. Aff., d**	**95% CI**	**Neg. Aff., d**	**95% CI**
Berman et al. (2008) Study 1	72	PANAS	1.05	0.93, 1.17	-	-
Berman et al. (2008) Study 2	24	PANAS	0.01	-0.31, 0.34	-	-
Hartig et al. (1991) Study 2	22	ZIPERS	1.10	0.69, 1.51	-1.15	-1.56, -0.73
Hartig et al. (1991) Study 2	22	OHS	0.72	0.34, 1.1	-	-
Hartig et al. (1999) Study 1	100	ZIPERS	0.43	0.35, 0.51	-0.71	-0.8, -0.63
Hartig et al. (2003)	112	ZIPERS	1.51	1.42, 1.6	-0.58	-0.65, -0.5
Ryan et al. (2010) Study 2	80	SVS	0.59	0.49, 0.69	-	-
Ryan et al. (2010) Study 3	97	SVS	0.76	0.67, 0.85	-	-
Sheets and Manzer (1991) Study 1	168	Affective Pleasure	0.68	0.63, 0.73	-	-
Sheets and Manzer (1991) Study 2	69	Affective Pleasure	1.07	0.94, 1.2	-	-
Ulrich et al. (1991)	120	ZIPERS F	-	-	-0.58	-0.66, -0.5
Ulrich et al. (1991)	120	ZIPERS A/A	-	-	-0.88	-0.96, -0.8
Ulrich et al. (1991)	120	ZIPERS PA	1.42	1.33, 1.51	-	-
Ulrich et al. (1991)	120	ZIPERS S	-	-	-0.21	-0.28, -0.13
Ulrich et al. (1991)	120	ZIPERS A/I	0.32	0.25, 0.4	-	-
Mayer et al. (2009) Study 1	76	PANAS	0.65	0.19, 1.11	0.15	-0.32, 0.61
Mayer et al. (2009) Study 2	62	PANAS	0.63	0.12, 1.14	0.06	-0.45, 0.57
Nisbet and Zelenski (2011) Study 1	73	PANAS	1.15	0.65, 1.65	-0.50	-1, -0.01
Nisbet and Zelenski (2011) Study 2	80	PANAS	1.45	0.95, 1.94	-0.49	-0.99, 0
Tennessen and Cimprich (1995)	36	POMS Total	-	-	-0.41	-1.13, 0.32
Tennessen and Cimprich (1995)	36	POMS D-D	-	-	-0.44	-1.17, 0.29
White et al. (2010) Study 1	80	Subj. Happiness	1.27	0.79, 1.76	-	-
Reeves et al. (2019)	68	PANAS	0.42	-0.06, 0.9	-	-
Stewart and Haaga (2018)	94	PANAS	0.47	0.06, 0.88	-0.56	-0.97, -0.15
Golding et al. (2018)	38	PANAS	0.87	0.2, 1.54	0.00	0, 0
McAllister et al. (2017)	144	PANAS	0.42	0.09, 0.75	-0.42	-0.75, -0.09
McAllister et al. (2017)	148	PANAS	-0.09	-0.41, 0.23	-0.52	-0.84, -0.2
Lee et al. (2009)	22	Subj. Comfort	2.59	1.46, 3.72	-	-
Park et al. (2007)	24	Subj. Comfort	3.43	2.17, 4.69	-	-
Park et al. (2007)	24	Subj. Calm	1.69	0.75, 2.62	-	-
van den Berg et al. (2003)	106	POMS D-D	-	-	-0.70	-1.08, -0.32
van den Berg et al. (2003)	106	POMS A-H	-	-	-0.82	-1.2, -0.44
van den Berg et al. (2003)	106	POMS T-A	-	-	-0.45	-0.83, -0.07
van den Berg et al. (2003)	106	OHS	0.63	0.24, 1.02	-	-
Tyrväinen et al. (2014)	154	PANAS	0.72	0.39, 1.05	-0.49	-0.82, -0.17
Tyrväinen et al. (2014)	154	SVS	0.22	-0.09, 0.54	-	-
Olafsdottir et al. (2018)	44	PANAS	1.03	0.4, 1.67	-0.72	-1.35, -0.09
Olafsdottir et al. (2018)	44	PANAS	0.86	0.24, 1.48	-0.64	-1.26, -0.02
Stigsdotter et al. (2017)	94	POMS T-A	-	-	-1.14	-1.54, -0.73
Stigsdotter et al. (2017)	94	POMS D-D	-	-	-0.12	-0.52, 0.29
Stigsdotter et al. (2017)	94	POMS A-H	-	-	-0.48	-0.89, -0.08
Stigsdotter et al. (2017)	94	POMS F-I	-	-	-0.16	-0.57, 0.24
Stigsdotter et al. (2017)	94	POMS C-B	-	-	-	-
Stigsdotter et al. (2017)	94	POMS V-A	0.37	-0.04, 0.77	-	-
Stigsdotter et al. (2017)	94	POMS Total	-	-	-0.54	-0.95, -0.14
Brown et al. (2013)	46	PANAS	-0.07	-0.65, 0.51	-0.44	-1.02, 0.14
Shin et al. (2011)	120	POMS T-A	-	-	-1.39	-1.75, -1.04
Shin et al. (2011)	120	POMS D-D	-	-	-1.01	-1.37, -0.65
Shin et al. (2011)	120	POMS A-H	-	-	-1.33	-1.69, -0.98
Shin et al. (2011)	120	POMS V-A	0.00	-0.36, 0.36	-	-
Shin et al. (2011)	120	POMS F-I	-	-	-1.55	-1.91, -1.19
Shin et al. (2011)	120	POMS C-B	-	-	-1.58	-1.93, -1.22
Beute and de Kort (2014) Study 2	60	Hedonic Tone	0	-0.51, 0.51	-	-
Bielinis et al. (2018)	54	POMS T-A	-	-	-0.57	-1.1, -0.04
Bielinis et al. (2018)	54	POMS D-D	-	-	-0.52	-1.06, 0.01
Bielinis et al. (2018)	54	POMS A-H	-	-	-0.43	-0.96, 0.1
Bielinis et al. (2018)	54	POMS F-I	-	-	-0.79	-1.33, -0.26
Bielinis et al. (2018)	54	POMS C-B	-	-	-0.71	-1.24, -0.18
Bielinis et al. (2018)	54	POMS V-A	0.65	0.1, 1.2	-	-
Bielinis et al. (2018)	54	PANAS	0.63	0.08, 1.18	-0.5	-1.04, 0.05
Geniole et al. (2016)	31	Pos. Mood	1.84	1.24, 2.43	-	-
Janeczko et al. (2020)	40	PANAS	-0.37	-1.01, 0.26	-0.28	-0.91, 0.35
Johansson et al. (2011)	20	NMS A/D	-	-	0.02	-0.6, 0.64
Johansson et al. (2011)	20	NMS A	-	-	-0.1	-0.72, 0.52
Liu et al. (2021)	30	POMS T-A	-	-	-1.78	-2.29, -1.27
Liu et al. (2021)	30	POMS D-D	-	-	-1.01	-1.52, -0.5
Liu et al. (2021)	30	POMS A-H	-	-	-0.88	-1.38, -0.37
Liu et al. (2021)	30	POMS F-I	-	-	-0.99	-1.5, -0.49
Liu et al. (2021)	30	POMS C-B	-	-	-1.45	-1.96, -0.95
Liu et al. (2021)	30	POMS V-A	1.98	1.36, 2.6	-	-
Lopes et al. (2020)	62	PANAS	0.92	0.39, 1.44	-0.82	-1.35, -0.3
Neale et al. (2021) Study 1	45	Hedonic Tone	0.64	0.21, 1.06	-	-
Takayama et al. (2014)	45	POMS T-A	-	-	-0.83	-1.24, -0.41
Takayama et al. (2014)	45	POMS D-D	-	-	-0.32	-0.74, 0.09
Takayama et al. (2014)	45	POMS A-H	-	-	-0.34	-0.75, 0.07
Takayama et al. (2014)	45	POMS F-I	-	-	-0.71	-1.12, -0.3
Takayama et al. (2014)	45	POMS C-B	-	-	-0.71	-1.12, -0.3
Takayama et al. (2014)	45	POMS V-A	0.96	0.52, 1.39	-	-
Takayama et al. (2014)	45	PANAS	0.24	-0.17, 0.66	-0.63	-1.05, -0.22
Brooks et al. (2017) Study 1	120	PANAS	0.27	-0.09, 0.63	-	-
Brooks et al. (2017) Study 2	58	PANAS	0.14	-0.37, 0.66	-0.42	-0.94, 0.09
Jiang et al. (2021)	17	Mood	1.65	0.53, 2.76	-	-
Lee et al. (2015a)	11	POMS T-A	-	-	-0.73	-1.57, 0.1
Lee et al. (2015a)	11	POMS D-D	-	-	-0.29	-1.12, 0.55
Lee et al. (2015a)	11	POMS A-H	-	-	-0.7	-1.54, 0.14
Lee et al. (2015a)	11	POMS V-A	0.66	-0.2, 1.52	-	-
Lee et al. (2015a)	11	POMS F-I	-	-	-0.83	-1.66, 0.01
Lee et al. (2015a)	11	POMS C-B	-	-	-0.51	-1.34, 0.33
Navalta et al. (2021)	10	Subj. Calm	1.09	0.15, 2.03	-	-
Navalta et al. (2021)	10	Subj. Comfort	1.3	0.34, 2.27	-	-
Nisbet et al. (2019)	65	PANAS	0.98	0.46, 1.49	-0.24	-0.75, 0.28
Pasca et al. (2021)	306	PANAS	6.47	5.91, 7.03	-7.5	-8.06, -6.95
Song et al. (2014)	17	POMS T-A	-	-	-0.61	-1.28, 0.06
Song et al. (2014)	17	POMS D-D	-	-	-0.57	-1.25, 0.1
Song et al. (2014)	17	POMS A-H	-	-	-0.57	-1.24, 0.11
Song et al. (2014)	17	POMS V-A	0.57	-0.12, 1.25	-	-
Song et al. (2014)	17	POMS F-I	-	-	-0.47	-1.14, 0.2
Song et al. (2014)	17	POMS C-B	-	-	-0.41	-1.08, 0.27
Song et al. (2014)	13	Subj. Comfort	1.55	0.68, 2.43	-	-
Ballew and Omoto (2018)	100	Pos. Emotions	0.58	0.18, 0.98	-	-
Bratman et al. (2015)	60	PANAS	-0.42	-0.93, 0.09	-0.52	-1.03, -0.01
Pretty et al. (2005)	40	POMS D-D	-	-	0.17	-0.45, 0.79
Pretty et al. (2005)	40	POMS A-H	-	-	0.18	-0.44, 0.8
Pretty et al. (2005)	40	POMS F-I	-	-	0.31	-0.31, 0.93
Pretty et al. (2005)	40	POMS C-B	-	-	0.39	-0.23, 1.01
Pretty et al. (2005)	40	POMS V-A	-0.26	-0.89, 0.36	-	-
Pretty et al. (2005)	40	POMS T-A	-	-	0.17	-0.45, 0.79
Koselka et al. (2019)	38	PANAS	0.65	0.54, 0.76	-	-

*Dash(–) indicates information not reported. For ZIPERS: F, Fear; A/A, Anger/Aggression; PA, Positive Affect; S, Sadness; A/I, Attentiveness/Interest. For POMS: T-A, Tension-Anxiety; D-D, Depression-Dejection; A-H, Anger-Hostility; V-A, Vigor Activity; F-I, Fatigue-Inertia; C-B, Confusion-Bewilderment. NMS, Negative Mood Scale. For NMS A/D, Anxiety/Depression, A, Anger*.

**Table 3:** Summary of meta-analysis results for various data subsets.

**Data subset**	**No. of studies**	***N***	**Pos. Aff., d**	**95% CI**	**Neg. Aff., d**	**95% CI**
Post-test results	49	3,201	0.86	0.75, 0.97	-0.67	-0.76, -0.58
Pre/post results	20	967	0.63	0.51, 0.75	-0.56	-0.64, -0.49
ZIPERS	4	354	0.94	0.66, 1.22	-0.62	-0.73, -0.5
PANAS	23	1,641	0.76	0.54, 0.98	-0.81	-1.09, -0.53
POMS	10	697	0.60	0.37, 0.83	-0.66	-0.73, -0.58
Immersion	29	1,467	0.85	0.75, 0.95	-0.68	-0.74, -0.62
Laboratory simulation	20	1734	0.82	0.58, 1.07	-0.65	-0.91, -0.39
Between-subjects	30	2,602	0.81	0.65, 0.98	-0.61	-0.76, -0.45
Within-subjects	19	599	0.90	0.76, 1.03	-0.75	-0.83, -0.67

Effect sizes require a degree of interpretation in application. Cohen (1988) suggests the following estimates for effect size d: 0.2 as a small effect, 0.5 as a medium effect, and 0.8 as a large effect. It is worth noting that in the same section, Cohen (1988) recognizes that the descriptive terms “small”, “medium”, and “large” are “relative, not only to each other, but to the area of behavioral science or even more particularly to the specific content and research method being employed in any given investigation” (Cohen, 1988, p. 25). In the absence of further guidance on effect size interpretation of the effects of nature on humans, Cohen's definitions have been adopted herein. The application of these definitions is further discussed in the Section 6.2.

Evidence of a large effect was found regarding the effect of natural environments on increasing positive affect, relative to the effect of urban environments (*d* = 0.86, 95*%CI* = 0.75, 0.97). The effect on reducing negative affect was of a medium to high order of magnitude (*d* = −0.67, 95*%CI* = −0.58, −0.76). These results lend validity to the primary hypothesis of this meta-analysis and to the claim of the biophilia hypothesis to be an emotional connection to nature.

Immersion in nature was identified to have a large effect (*d* = 0.85, 95*%CI* = 0.75, 0.95) and laboratory simulation a medium to large effect (*d* = 0.82, 95*%CI* = 0.58, 1.1) with regards to positive affect. A similar relationship was found for negative affect, with immersion resulting in a medium to large effect (*d* = −0.68, 95*%CI* = −0.62, −0.74) and laboratory simulation a medium effect (*d* = −0.65, 95*%CI* = −0.39, −0.91).

Although the central estimate for the effects of both immersion and laboratory exposure are comparable, the 95% confidence interval for the effects of immersion is much narrower and almost entirely sits within the definition of “large effect”. Therefore, these results appear to support the hypothesis that an increasing level of sensory connection to nature results in an increased benefit. This result in particular has implications for biophilic design and is further explored in the Section 6. This result aligns with that of Mayer et al. (2009), which indicated that “virtual” nature was less effective in helping participants reflect on a life problem and less effective in causing beneficial psychological effects. Note that the term “virtual” was used to indicate laboratory simulation of nature through videotapes, and was not related to more advanced concepts of virtual, interactive, and immersive environments which have also been explored (Valtchanov et al., 2010).

**Figure 2:**
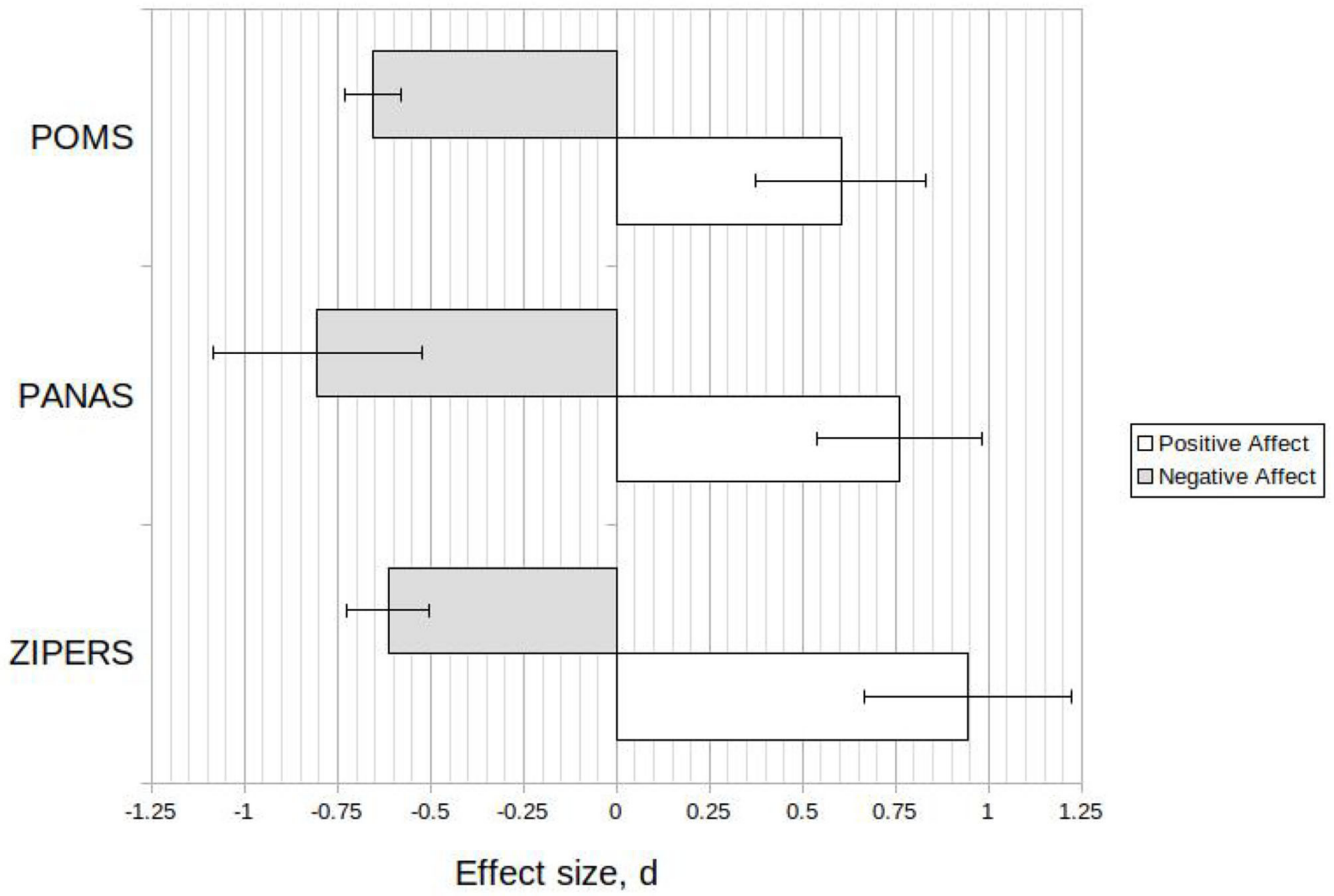
Calculated effect sizes, d, for common outcome measures (Mean ± 95%CI).

A comparison of outcome measures was conducted as presented in Figure 2. The ZIPERS outcome measure for positive affect resulted in the highest effect size (*d* = 0.94, 95*%CI* = 0.66, 1.2). The 95% confidence interval for ZIPERS was wide due to only 4 studies (total participants n = 354) utilizing the measure.

POMS displayed a low to moderate effect size for positive affect (*d* = 0.60, 95*%CI* = 0.37, 0.83) and a moderate effect size for negative affect (*d* = −0.66, 95*%CI* = −0.58, −0.73) affect. This result supports the use of POMS as a measure of mood disturbance (McNair et al., 1971). The result also implies that the POMS is able to weakly measure positive affect (through a single item).

The PANAS measure displayed medium to large effect sizes for both positive affect (*d* = 0.76, 95*%CI* = 0.54, 0.98) and negative affect (*d* = −0.66, 95*%CI* = −0.58, −0.73).

**Figure 3:**
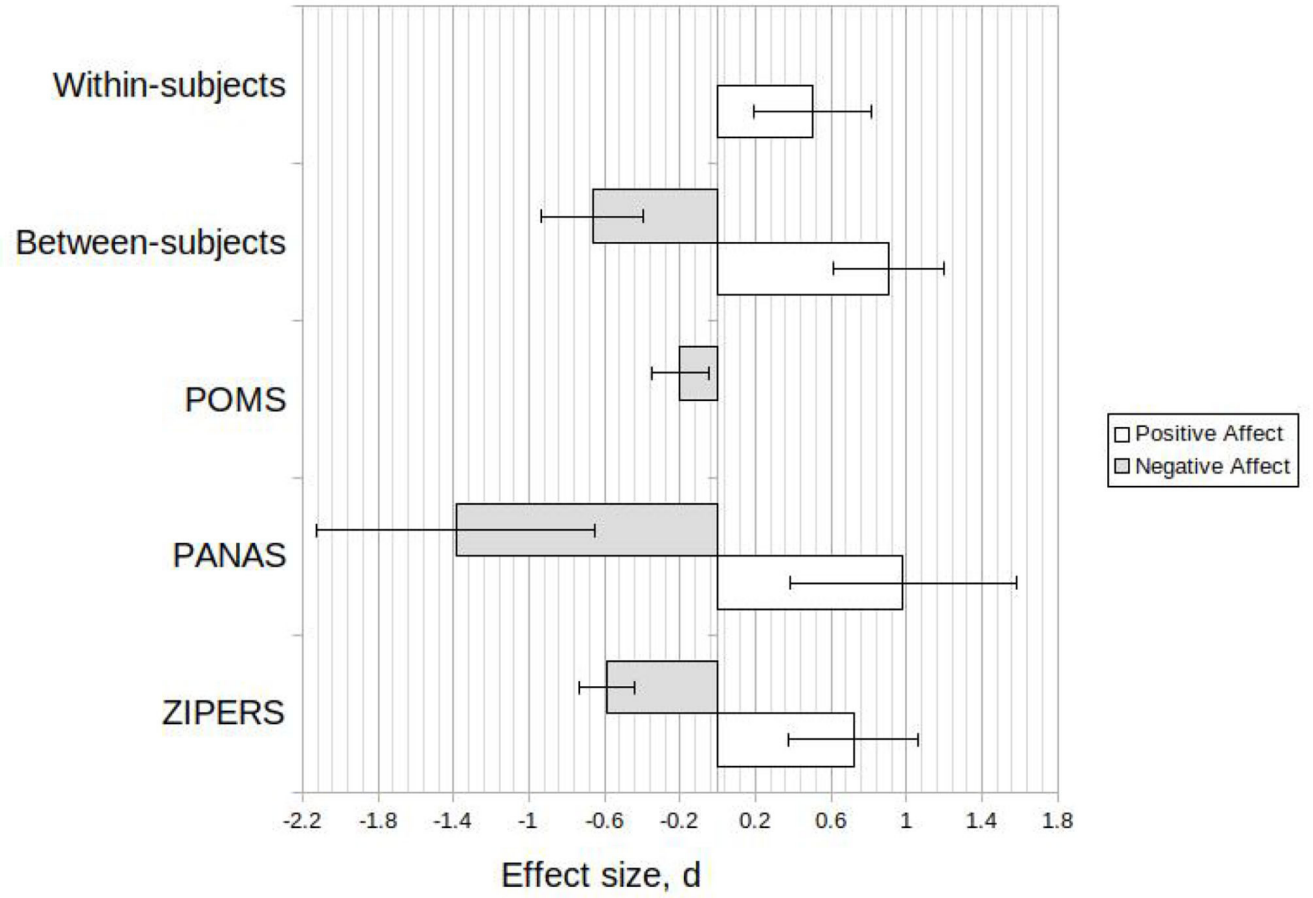
Calculated effect sizes, d, for laboratory simulation only data set and various experimental methods and outcome measures (Mean ± 95%CI).

Further investigation was conducted on a dataset which included only studies which utilized laboratory simulation in their method. This investigation was conducted in direct response to the two secondary objectives of this study. The relative effect of outcome measure showed a similar relationship to the combined data however, with a smaller magnitude (refer Figure 3).

Use of PANAS resulted in the largest effect sizes for positive affect (*d* = 0.98, 95*%CI* = 0.38, 1.6) and negative affect (*d* = −1.4, 95*%CI* = −0.65, −2.1). Within-subjects study design showed considerable variation as the sample size included only 4 studies with a combined total of 195 participants. The effect size of two of these studies (Berman et al., 2008; Brown et al., 2013) was close to zero for positive affect. Both of these studies also used PANAS as the outcome measure for positive affect.

#### 5.4.3. Pre/post Difference Dataset

The previous analysis used post-test data only. The quality of this data was lower than pre/post data as no control was made for pre-test conditions. An analysis of pre/post data was therefore conducted to assess the change that exposure to natural and urban environments induces in participants. A medium effect size (*d* = 0.63, 95*%CI* = 0.51, 0.75) for positive effect was determined. A medium effect size (*d* = −0.59, 95*%CI* = −0.45, 0.73) was also determined for negative effect. The pre/post difference data demonstrate similar behavior to the post-test only data, which provides a level of credibility to the lower quality post-test data.

#### 5.4.4. Confounding Effects

The analysis revealed that confounding effects exist which may challenge the initially apparent conclusions associated with the reported results. All studies which used ZIPERS as an outcome measure also only reported post-test statistics. Similarly, all except a single study which used POMS as an outcome variable reported pre/post mean and standard deviation data. Further investigation of the four studies which used ZIPERS revealed that participants were exposed to a stressor in 3 of the 4 studies (Hartig et al., 1991, 2003; Ulrich et al., 1991). Use of a stressor may result in a greater magnitude of mood induction due to environment (Hartig et al., 1999). Hence, the resultant effect size associated with the ZIPERS outcome measure may be related to the experimental method rather than the outcome measure. Recommendations on outcome measures for future use are included in the Section 6.

## 6. Discussion

### 6.1. Primary Findings

#### 6.1.1. Body of Research

The body of research identified through the literature search was substantial, although of varying quality. Specifically, further information regarding the test environments and type of reported data are deemed to be critical. Additional information on each environment studied would allow further analysis on the effect of types of natural and urban environments. This information could include additional images of each environment and descriptions by the study authors, potentially appended to each article or stored on a digital repository. Further data on test environments would allow the body of research to advise on particular properties or patterns of nature (Kahn and Hasbach, 2012; Downton et al., 2016; Roös, 2021) that are effective.

The quality of reported quantitative result data was variable. The availability of pre-treatment and post-treatment mean and standard deviation data was uncommon. It is recommended that pre/post mean and standard deviation data is reported in all studies, along with the calculated difference mean and standard deviation. These data should be presented for each test environment. The reporting of such data is especially important for within-subjects designs where the correlation between pre- and post- measures is not provided. It is noted that provision of such data is not relevant in demonstrating the significance of results, but serves iterative science.

#### 6.1.2. Effects of Environment

The results indicate that the effects of environment on positive and negative affect are medium to large. This outcome appears to support the proposed emotional dimension of the biophilia hypothesis, and through this, the concept of biophilic design. This result achieves the primary objective of this study and the hypothesis associated with this objective. However, the pathway by which this benefit is realized is unclear. Furthermore, the increases to positive affect are of comparable magnitude to the reductions of negative affect. This may indicate that natural environments are beneficial not only in supporting recovery from physiological stress (Corazon et al., 2019), but supporting recovery from a negatively perturbed affective state also.

Insofar as the structure of the biophilia hypothesis was defined in the Section 2 of this paper, the results appear to evidence support for the hypothesis. However, although the results support the biophilia hypothesis, there are a number of theories which would also indicate an affirmative result. These theories were briefly raised in the Section 2 of this paper, using the concept of “psychoterratic typologies” (Albrecht, 2019). The diversity of the theoretical landscape is significant as it requires the pathway by which the evidenced benefits are realized to be identified. This study of pathway can then be used to distinguish effects and evidence both the biophilia hypothesis and biophilic design theory in contrast to evidencing any number of other related theories. The critical examination of Joye and De Block (2011) discussed in the Section 2 is particularly relevant, as without a more specific definition of the many theoretical frameworks, it is difficult to distinguish what a result may imply in relation to the broad body of theories.

The reduction in magnitude of negative affect invites a parallel to Stress Recovery Theory (Ulrich, 1979, 1981; Ulrich et al., 1991). There indeed may be an emotional component to Stress Recovery Theory, a finding which supports the proposed affective/arousal response model of Ulrich (1983). That is, exposure to a natural environment enables more rapid recovery of both emotions and stress from a negative perturbation. The model of Ulrich (1983) also presents an attractive parallel for the potential response pathways of the biophilia hypothesis, with both a pre-cognitive and post-cognitive component to human response to the environment. However, this proposed model requires further, specific data to be confirmed, especially around the effects of culture and experience on the post-cognition state. It is recommended that the PANAS outcome measure for negative affect is used in concert with stress outcome variables (Corazon et al., 2019) in further studies regarding the effects of environment on stress and affective recovery.

The integrated model of Kaplan (1995) and Han (2001) uses a “resource inefficiency” framework to combine the effects of nature on emotion and various definitions of attention and stress. The clear impacts on emotions revealed by the results may indicate support for this framework. However, simultaneous study of emotion, stress, and attention would provide a clearer understanding of the integrated framework.

When interpreting the significance of these results with respect to biophilic design, it is also critical to note that the results presented herein compare natural and urban environments. However, the application of biophilic design only carries benefits relative to the same environment were biophilic design not applied. Hence, the dominant applied design practice in each case of biophilic design must be used as a comparator. This renders determination of an effect difficult. Furthermore the magnitude of the relative effect of biophilic design will be less than the effect sizes reported herein. Studies such as Sheets and Manzer (1991), Lee et al. (2015b), and Yin et al. (2018) provide insight into the effects of what may be considered applied biophilic design with appropriate comparator environments. The use of a custom and undocumented Biophilic Interior Design Index by Yin et al. (2018) to “objectively rate each indoor environment based on the biophilic quality of the environment” (Yin et al., 2018, p. 257) opens another potential question; that is, is the effect of the environment on occupants the only way to determine the quality of applied biophilic design, or can the application of biophilic design be determined by inspection? Again, referencing the model of Ulrich (1983), subjective assessment of biophilic design by occupants/participants would form part of the post-cognitive pathway only. This would negate the pre-cognitive genetic-based effects proposed by the affective/arousal response model.

#### 6.1.3. Sensory Connection

A comparison of the effects of immersion in environments with laboratory simulation of environments lends itself to a discussion around the concept of sensory connection to nature and how study participants were connected to the study environments. The key difference between environment type was the level of sensory connection available to study participants—immersion enables full sensory connection whereas laboratory simulation is a partial, simulated connection. Immersion in natural environments demonstrated a larger effect on emotion in comparison with laboratory simulation of natural environments. This result achieved the first further objective of this study and its relevant hypothesis, as an increase in the affective benefits of environment was linked to an increase in sensory connection.

It is proposed that the effects of these two states of sensory connection with nature may be considered as two points on a continuous scale of affective response to the environment, with immersion in nature forming the upper bound of the scale. Effects of implementations of biophilic design would also be variously located on this scale, depending on their effectiveness in benefiting occupants. A prospective designer implementing biophilic design would therefore be advised to seek to increase the level of sensory connection to nature through the design, in order to increase the effectiveness of the design in supporting a positive emotional state.

The level of immersion tested by the studies involved only passive contact with nature; typically sitting and viewing nature while immersed, or walking along a predefined path and observing nature. Active interaction or participating in nature, such as elements of “interaction patterns” (Kahn and Hasbach, 2012), were not studied. It may be that interaction along these patterns will garner greater emotional benefits for humans that the results reported herein. Therefore, the benefits of nature discussed in this meta-analysis should present a starting point only from which meaningful interaction with nature will exceed. As an example, studies on gardening and community gardening show a wide range of positive psychophysical and social aspects (van den Berg and Custers, 2011; Poulsen et al., 2014; Chang et al., 2016; Lee, 2017; Lin et al., 2018; Stoltz and Schaffer, 2018). This level of interaction, and it's relationship to the passive exposure methods studied in herein, may be explored to further develop the narrative around benefits of experience of nature and hence biophilic design.

#### 6.1.4. Experimental Methods

The meta-analysis did not result in widespread clarity regarding use of certain experimental methods for future investigations into the biophilia hypothesis and biophilic design. Study design favors immersion in nature, likely due to the increased sensory connection and therefore potential for connection with nature. This was discussed in the previous section. Use of the PANAS scales for positive and negative affect indicated a significant effect. As a large number (23) of studies used this outcome measure, and this measure displays sensitivity to both positive and negative affect, use of the PANAS scale is recommended for further research into the effect of environment on affect. As previously mentioned in this discussion, the use of the PANAS negative affect scale outcome measure is recommended as a measure of negative affect in concert with outcome variables associated with stress. ZIPERS was demonstrated to be a sensitive outcome measurement tool. However, several confounding effects potentially reduce the clarity of the ZIPERS dataset. As discussed in the Section 5, this is largely due to a single cohort of researchers utilizing ZIPERS, with accompanying methodological similarities between the ZIPERS studies. The results indicate that POMS is a sensitive measure of mood disturbance for a range of methodologies including laboratory simulations of nature.

### 6.2. Limitations

There are several limitations of the reported meta-analysis. The post-treatment dataset was considered of moderate quality as the pre-test condition was not accounted for in the analysis. The analysis of the pre/post dataset was conducted to directly address this potential limitation, and identified that the results indicated by the post-treatment data set were valid, although potentially overestimated.

Limitations associated with the method of the meta-analysis concern the interpretation of effect sizes, and the comparison between environments. The interpretation of effect sizes originally suggested by Cohen (1988) was used when interpreting results. However, it is unclear whether there is a correlation between numerical effect sizes and wellbeing of participants. For example, does a large effect size correspond to a “large” increase in positive emotion as experienced by a study participant? Further exploration of the correlation between quantitative data and wellbeing outcomes is required to bridge this gap.

The analysis conducted sought to compare the effects of natural and urban environments. It is anticipated that this comparison maximizes observed effects, that is, natural environments are rich in elements that support positive affect and reduce negative affect, and urban environments are rich in elements which reduce positive affect and support negative affect. Hence, comparing the effects of these two environments may maximize the reported effects and their significance.

## 7. Conclusions

The primary objective of this meta-analysis was to determine the relative effect of natural environments on positive and negative affect. This objective was constructed in the context of the biophilia hypothesis. This theory informed the study hypothesis, that exposure to natural environments would positively impact affective state more than urban environments. The study hypothesis was confirmed, with natural environments found to have a medium to large effect on increasing positive emotion and decreasing negative emotion.

Support for the emotional dimension of the biophilia hypothesis was ascertained. Furthermore, the results appeared to provide evidence for the affective/arousal response model of Ulrich (1983). However, further study of human response pathways associated with the biophilia hypothesis is required to enable the biophilia hypothesis and its effects on humans to be singled out from the broad theoretical landscape considering the relationship between humans and their environment.

Two secondary objectives were also achieved. Immersion in environments was found to have a larger effect than laboratory simulations of environments, confirming the study hypothesis. This finding had implications for biophilic design by encouraging the use of increased sensory immersion in the biophilic design paradigm.

The impact of study methodology on outcomes was less clear. Use of the PANAS scale for positive and negative affect indicated a significant effect. A large number (23) of studies used PANAS as an outcome measure. As PANAS displays sensitivity to both positive and negative affect, use of the PANAS scale is recommended for further research into the effect of environment on affect. The ZIPERS scales and POMS scales also indicate sensitivity. However, there are confounding effects associated with the ZIPERS dataset. Use of the PANAS negative affect scale outcome measure is recommended as a measure of negative affect in concert with outcome variables associated with stress.

## Author's Note

This paper is part of the activities of the ongoing research under an approved PhD Candidature at Deakin University, School of Architecture and Built Environment, titled: The role of individual differences in the biophilia hypothesis and implications for biophilic design by JG.

## Data Availability Statement

The data that support the findings of this study are available from the corresponding author, JG, upon reasonable request.

## Author Contributions

JG: data collection, analysis, interpretation, and manuscript preparation. AS, PR, AW: data collection and critical revision. All authors contributed to the article and approved the submitted version.

## Funding

The authors acknowledge the financial support by the Live+Smart Research Laboratory, Deakin University for assistance in the publication of this article, as well as providing its infrastructure for research.

## Conflict of Interest

The authors declare that the research was conducted in the absence of any commercial or financial relationships that could be construed as a potential conflict of interest.

## Publisher's Note

All claims expressed in this article are solely those of the authors and do not necessarily represent those of their affiliated organizations, or those of the publisher, the editors and the reviewers. Any product that may be evaluated in this article, or claim that may be made by its manufacturer, is not guaranteed or endorsed by the publisher.
